# Book Reviews

**Published:** 1898-07

**Authors:** 


### Book Reviews.

---

PROCEEDINGS OF THE AMERICAN MEDICO-PSYCHOLOGICAL ASSOCIATION at the Fifty-third Annual Meeting, held at Baltimore, May 11-14, 1897. C. B. Burr, M. D., Secretary, Flint, Michigan. Published by American Medico-Psychological Association. 1897.

This book contains the proceedings of this association at its fifty-third annual meeting, together with a list of members of the association, as well as the papers and discussions read and held at the meeting. It is a well-edited volume and is full of useful material. A book so well printed and containing so many papers of interest deserves a binding more substantial than paper. Every alienist should keep files of these transactions. The association is the oldest special society in this country.

Some of the papers offered, deserve special commendation, particularly those of Dr. Hurd, of Buffalo, on Clinical aspects of auto-intoxication; Dr. Adolf Meyer, on Demonstration of various types of changes in the giant cells of the paracentræ labule, which is accompanied by three handsome full-page colored plates of ganglion cells; Report of a case of progressive dementia—clinical and pathological—by Charles K. Mills and Dr. Mary A. Schively, of Philadelphia; Industrial employment of the insane, by Dr. G. Alder Blumer; Insanity following surgical operations, by Dr. Richard Dewey; The after-care of the insane, by Henry R. Steadman; Canadian jurisprudence of insanity, by Dr. Daniel Clark; The constructive forces, by Dr. Ralph L. Parsons; The psychology of insane delusions, by Dr. W. L. Worcester. There are also able papers by other members of the society.

---

FAT AND BLOOD. An essay on the treatment of certain forms of neurasthenia and hysteria. By S. WEIR MITCHELL, M. D., member of the National Academy Sciences; Physician to the Orthopedic Hospital and Infirmary for nervous diseases, etc. Duodecimo, pp. 177. Seventh edition. Philadelphia: J. B. Lippincott Company. 1898.

Readers of the JOURNAL are already familiar with Dr. Mitchell's famous essay on fat and blood and need, therefore, no introduction to the same. The fact that seven large editions have been published of this essay shows deep-seated interest in this subject, and the results of reading such an admirable essay must surely not be without reward.

The chapter on massage has been rewritten and the whole work carefully revised under the direction of the author's son, Dr. John K. Mitchell.

The book is neatly printed and bound by the Lippincotts.

---

The Elements of Clinical Diagnosis. By Professor Dr. G. Klemperer, Professor of Medicine at the University of Berlin. First American from the seventh (last) edition. Duodecimo, pp. 292, with 61 illustrations. Authorised translation by Nathan E. Brill, A. M., M. D., Adjunct Attending Physician Mount Sinai Hospital, New York City, and Samuel M. Brickner, A. M., M. D., Assistant Gynecologist Mount Sinai Hospital, out-patient department. New York: The Macmillan Company, 66 Fifth avenue. 1898.

The author of this little work is a practical diagnostician and his book contains the rules governing the clinical diagnosis of medical cases in his clinic at Berlin. Many Americans have taken his course and they will be pleased to have access to the work through this translation. In seven years it has gone through seven German editions, but now appears for the first time in English. The woik is a concise setting forth of the elements of clinical diagnosis and will be found most helpful to the student. It is well printed and contains a number of useful illustrations. The author through the several editions has kept it abreast of medical progress, even to adding a chapter on the Röntgen rays.

---

Transactions of the Medical Society of the District of Columbia, from March, 1896, to January, 1897. Editing committee: W. W. Johnston, M. D., Geo. M. Kober, M. D., and Jas. D. Morgan, M. D. Volume I. 1897.

This excellent society has issued a large and interesting volume full of scientific material that shows careful editing. The absence of an index, however, renders it well-nigh impossible to utilise it. We trust this defect may be remedied in subsequent volumes. The example of this society in preserving and publishing its work deserves imitation by other city medical organisations.

---

A Manual of Instruction in the Principles of Prompt Aid to the Injured. Including a chapter on Hygiene and the Drill Regulations for the Hospital Corps, U. S. A. By Alvah H. Doty, M. D., Health Officer of the Port of New York, etc. Duodecimo, pp. xvi.—302. New York: D. Appleton & Co. 1898.

We have had occasion heretofore when earlier editions were under consideration to speak well of Dr. Doty's little book, and desire now to reaffirm our former judgment after a further examination of its merits. It is a timely act on the part of the publishers to put forth a reprint of the work at this time when the information and hints it contains will prove so valuable. Every medical officer joining the medical staff of the volunteer army should provide himself with a copy and it will do him no harm to study it carefully. It contains much, too, that officers of the line should be familiar with and we doubt not they will be larger purchasers of the work. Its convenient size makes it easily carried in the field.

